# Immunohistochemical Detection of *Propionibacterium acnes* in the Retinal Granulomas in Patients with Ocular Sarcoidosis

**DOI:** 10.1038/s41598-017-15710-0

**Published:** 2017-11-09

**Authors:** Kenji Nagata, Yoshinobu Eishi, Keisuke Uchida, Kazuhito Yoneda, Hiroki Hatanaka, Toru Yasuhara, Maho Nagata, Chie Sotozono, Shigeru Kinoshita

**Affiliations:** 1 Department of Ophthalmology, Kyoto Prefectural University of Medicine, 465 Kajii-cho, Hirokoji-agaru, Kawaramachi-dori, Kamigyo-ku, Kyoto 602-0841 Japan; 2 Department of Human Pathology, Tokyo Medical and Dental University, 1-5-45 Yushima, Bunkyo-ku, Tokyo 113-8510 Japan; 3 Department of frontier medical science and technology for ophthalmology, Kyoto Prefectural University of Medicine, 465 Kajii-cho, Hirokoji-agaru, Kawaramachi-dori, Kamigyo-ku, Kyoto 602-0841 Japan

## Abstract

The etiology of sarcoidosis is still obscure; however, Mycobacteria and *Propionibacterium acnes* are considered the most implicated etiological agent for sarcoidosis. To investigate whether *P. acnes* is an etiological agent for sarcoid uveitis, we analyzed the frequency of *P. acnes* detected within the biopsied retinas from patients with ocular sarcoidosis by immunohistochemistry with a *P. acnes*-specific monoclonal antibody (PAB antibody). Eleven patients (12 eyes) with sarcoid uveitis were enrolled in this study. Eight patients with rhegmatogenous retinal detachment, two patients with non-sarcoid uveitis, and two patients with vitreoretinal lymphoma were enrolled as controls. In the sarcoidosis group, granulomas were mainly observed in the inner retinal layer filled with CD4+ cells and CD68+ cells, indicating the Th1 immune response. *P. acnes*, identified as round bodies that reacted with the PAB antibody, were present in 10/12 samples (83%) from 9/11 patients (82%) with sarcoidosis. These round bodies were scattered within the retinal granulomas mainly in the inner retinal layer. In the control group, no round bodies were detected. Our results suggested that *P. acnes* could be associated with sarcoid uveitis. We hypothesize that sarcoid granulomas may be formed by a Th1 immune response to *P. acnes* hematogenously transmitted to the retina.

## Introduction

Sarcoidosis is a multisystemic granulomatous disorder characterized by the presence of noncaseating granulomas in the involved organs^1,2^. This disorder commonly affects young and middle-aged adults who present with bilateral hilar lymphadenopathy, pulmonary infiltration, and ocular and skin lesions^2^. The liver, spleen, lymph nodes, heart, salivary glands, nervous system, muscles, and other organs may also be involved.

The etiology of sarcoidosis is still obscure; however, sarcoidosis is thought to be triggered by either infectious agents or exposure to environmental substances in patients with various genetic factors, such as the *HLA-DRB1* gene^3–7^. Mycobacteria are considered to play a major etiologic role in sarcoidosis in the United States and Europe^8–14^. On the other hand, *Propionibacterium acnes* is considered the most implicated etiological agent for sarcoidosis in Japan because it has been isolated at a high ratio from systemic sarcoid lesions by bacterial culture^15,16^. Bacterial culture has shown that *P. acnes* is present in up to 78% of biopsied lymph nodes from Japanese patients with sarcoidosis, while only 21% of non-sarcoidosis patients showed positive results^16^. Moreover, higher number of *P. acnes* genomes has been detected in sarcoid lymph nodes than in control samples using quantitative polymerase chain reaction (PCR)^17,18^. Furthermore, Negi, *et al*. reported that *P. acnes* was present within sarcoid granulomas in 74% of video-assisted thoracic surgery lung samples, 48% of transbronchial lung biopsy samples, 88% of Japanese lymph node samples, and 89% of German lymph node samples by immunohistochemical analysis using a *P. acnes*-specific monoclonal antibody (PAB antibody)^19^. Lipoteichoic acids are membrane-anchored molecules in the cell envelopes of gram-positive bacteria^20–24^. PAB antibody was developed to react specifically with the cell-membrane-bound lipoteichoic acid of *P. acnes* ^19^. In addition, *P. acnes* can induce granulomatous inflammation in the murine lung^25^. These data imply that *P. acnes* is a pathogenic bacterium that may cause systemic sarcoidosis. However, only a few reports have demonstrated the presence of *P. acnes* in ocular tissue in patients with ocular sarcoidosis^26,27^. For example, Yasuhara *et al*. showed that *P. acnes* or *P. granulosum* DNA was present in the vitreous fluid of patients with sarcoidosis-associated uveitis^26^. To the best of our knowledge, no reports have clearly demonstrated the presence of *P. acnes* in the retina.

To diagnose systemic sarcoidosis, the most reliable method is analysis of biopsied specimens; the same is thought to be true in ocular sarcoidosis. Indeed, retinal biopsy or chorioretinal biopsy is thought to provide useful information for the diagnosis of uveitis^28–30^. Moreover, severe vitreous opacity, macular edema, and/or epiretinal membrane are often accompanied by posterior uveitis with sarcoidosis as the cause of impaired visual acuity^31,32^. For these cases, pars plana vitrectomy (PPV) is beneficial for improving visual acuity. We have some cases performed a retinal biopsy during PPV to diagnose sarcoidosis.

In the present study, we analyzed the frequency of *P. acnes* detected within the biopsied retinas from patients with ocular sarcoidosis by immunohistochemistry (IHC) with PAB antibody in order to prove possible involvement of *P. acnes* in the pathogenesis of ocular sarcoidosis.

## Results

### HE staining

**Figure 1:**
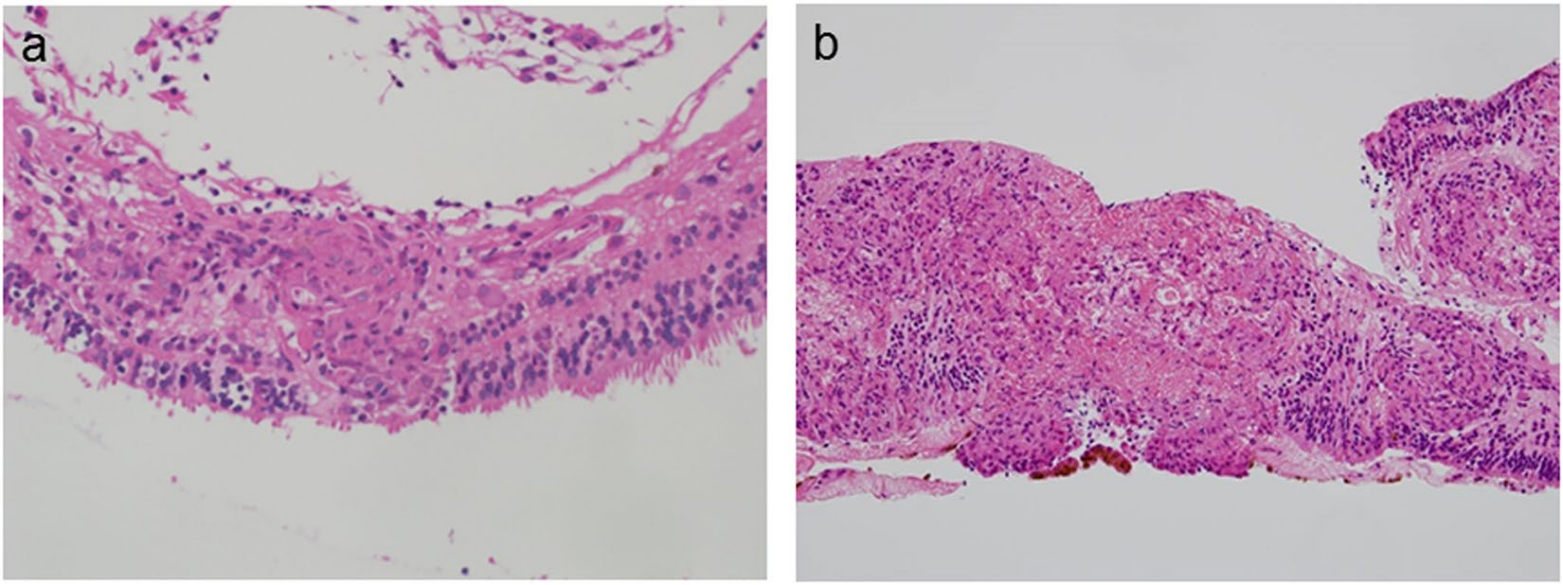
Hematoxylin and eosin staining of retinal samples from patients with sarcoidosis. (**a**) Granuloma was observed in the retinal sample. The granuloma existed mainly in the inner retinal layer (original magnification, 400×). (**b**) Large granulomas were detected in both the inner and outer retinal layers. The layered structure of the retina was disrupted by inflammation (original magnification, 200×).

Although the retinal samples were extremely small, all samples were sufficiently enough to be examined by HE staining. Histopathological examination of retinal specimens in this study confirmed the diagnosis in all 11 cases with sarcoidosis. In the sarcoidosis group, noncaseating epithelioid cell granuloma was observed in all cases. Granulomas were mainly located in the inner retinal layer (Fig. 1). In some severe cases, large granulomas were detected in both the inner and outer retinal layers, resulting in disruption of the layered structure of the retina, including the photoreceptor (Fig. 1). There were no cases with granuloma presenting only in the outer retinal layer. These findings indicated that the granulomas originated in the inner retinal layer and that the large granulomas had been growing outward into the surrounding retina, disrupting the retinal layered structure. In patients with RRD, no granulomas were observed. In patients with non-sarcoid uveitis and vitreoretinal lymphoma, infiltration of inflammatory cells was observed.

### *P. acnes* detection

**Figure 2:**
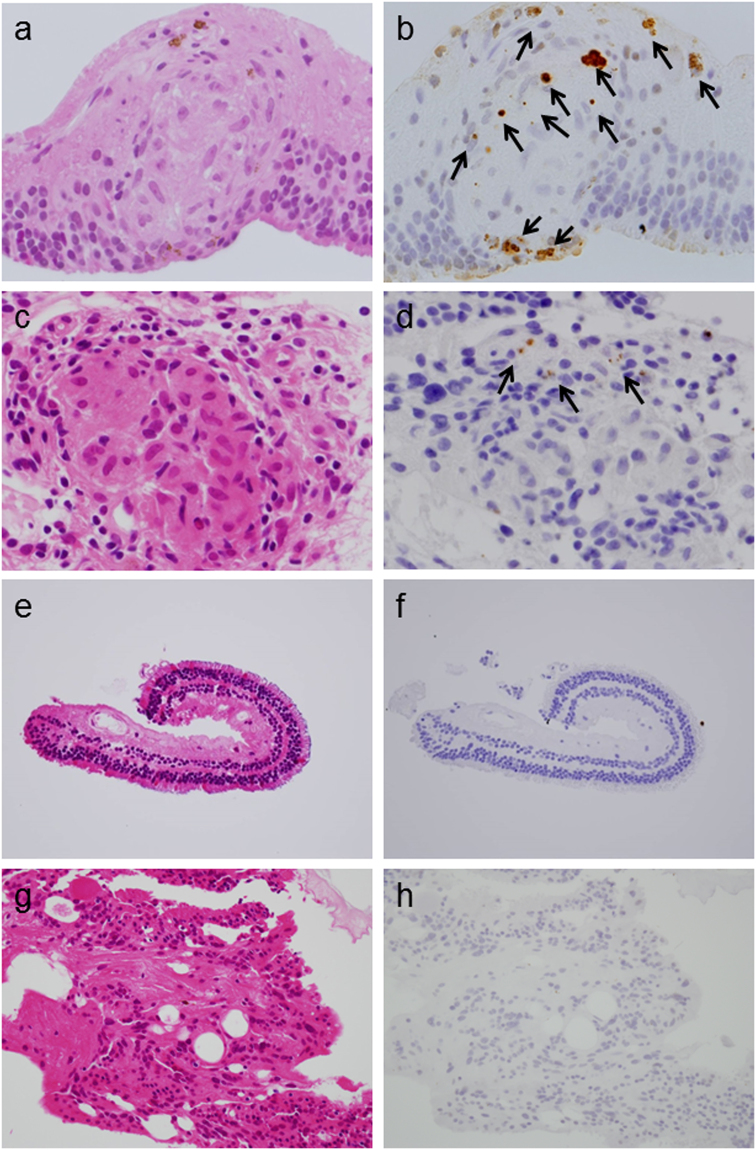
*P. acnes* within the retina. Hematoxylin and eosin staining and immunohistochemistry with PAB antibody are shown pairwise for retinal samples from patients with sarcoidosis (**a**–**d**), patients with rhegmatogenous retinal detachment (RRD) (**e,f**), and patients with acute retinal necrosis (**g**,**h**). (**a**,**b**) Many round bodies detected by the PAB antibody were observed in the granuloma of the retina (indicated by the arrows). These were divided roughly into small- and large-sized forms. Large spheroidal bodies were sparsely distributed. (**c**,**d**) Some small round bodies were sparsely observed in mature granulomas (indicated by the arrows). (**e**,**f**) Any round bodies detected by PAB antibody were not observed in the retina from patients with RRD. (**g**,**h**) Severe infiltration of inflammatory cells was observed in the retinal sample from a patient with acute retinal necrosis. However, no round bodies showing positive staining with the PAB antibody were observed in the retinal sample.

Round bodies immunohistochemically detected by PAB antibody were observed in 10/12 samples (83%) from the 9/11 patients (82%) with sarcoidosis, demonstrating the presence of *P. acnes* at these frequencies (Table 1). Round bodies were localized within the granulomas and in lesions infiltrated by inflammatory cells (Fig. 2), indicating that *P. acnes* was associated with the formation of granulomas and the resulting inflammation. Many retinal granulomas contained small- (0.5~2 µm in diameter) and/or large-sized (3~5 µm in diameter) forms of round bodies. In three of ten samples (30%) had round bodies detected by PAB antibody, large spheroidal bodies were sparsely detected using PAB antibody. The numbers of round bodies in the retinal sample varied among samples. In six of ten samples (60%), round bodies were sparsely detected. Similar to lung granulomas, immature granulomas tended to include more round bodies than did mature granulomas, suggesting that *P. acnes* may be related to the early stages of granuloma formation. In five of ten samples (50%), some round bodies were aggregated as though some granuloma-forming cells were filled with the bacterium. In contrast to the sarcoidosis group, *P. acnes* was undetectable in all control samples (Fig. 2). Significant difference in the prevalence of *P. acnes* was observed between the sarcoidosis group and the control group (*P* < 0.0001).

**Table 1:** Frequency of *P. acnes* detected by PAB antibody in retinal samples.

Disease	Total number of patients	Total number of eyes	Number (%) of patients with *P. acnes* detected in retina	Number (%) of eyes with *P. acnes* detected in retina
Sarcoidosis	11	12	9 (82)	10 (83)
RRD	8	8	0	0
Non-sarcoid uveitis	2	2	0	0
Vitreoretinal lymphoma	2	2	0	0

RRD: rhegmatogenous retinal detachment.

### Inflammatory reaction to form granulomas

**Figure 3:**
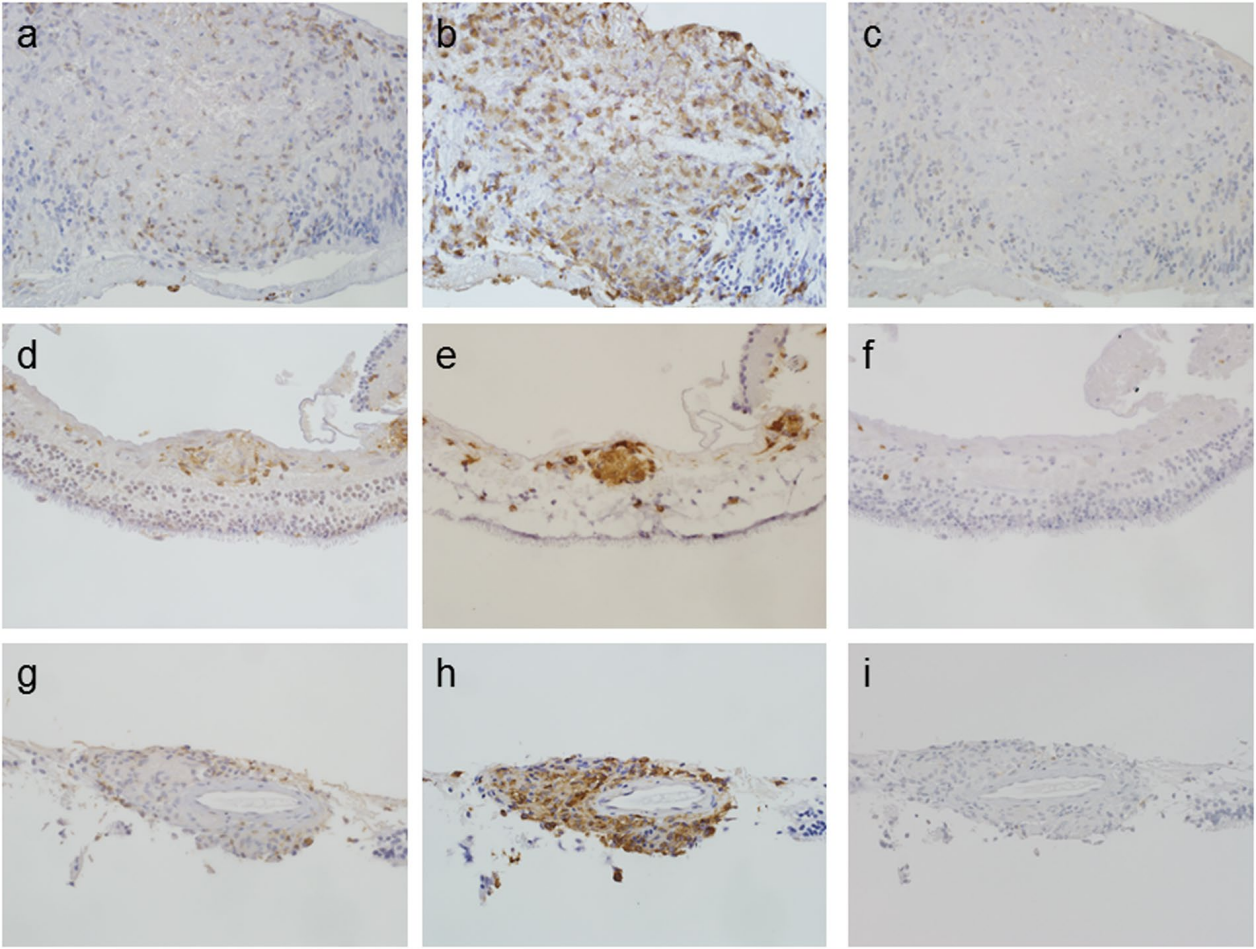
Immunohistochemistry with CD4, CD8, and CD68. (**a**,**d**,**g**), Anti-CD4 antibodies; (**b**,**e**,**h**), anti-CD68 antibodies; (**c**,**f**,**i**), anti-CD8 antibodies. (**a**–**c**), Large granulomas in the retinal sample were comprised of CD4+ cells (**a**) and CD68+ cells (**b**). CD8 + cells were rarely detected (**c**) (original magnification, 400×). (**d**–**f**), A small granuloma comprised of CD4+ cells (**d**) and CD68+ cells (**e**) was located in the inner retinal layer. CD8 + cells were rarely detected (**f**) (original magnification, 400×). (**g**–**i**), Inflammatory cells infiltrating around a blood vessel were detected and were positive for CD4 (**g**) and CD68 (**h**) but negative for CD8 (**i**) (original magnification, 400×).

Granulomas were mainly comprised of CD4+ cells and CD68+ cells (Fig. 3), indicating that the granulomas were formed by accumulation of CD4+ T lymphocytes and macrophages. These cells were primarily located in the inner retinal layer (Fig. 3), consistent with the presence of granulomas in this layer of the tissue. CD8+ cells were rarely observed in the retinal samples (Fig. 3), suggesting that the formed granulomas were not the result of cytotoxic T-lymphocyte accumulation. In some cases, these inflammatory cells, which were positive for CD4 or CD68, infiltrated conspicuously in the perivascular area in the inner retinal layer (Fig. 3), implying that the inflammatory reactions may involve retinal blood vessels.

## Discussion

The pathogenesis of ocular sarcoidosis is not yet fully understood. In the present study, we clearly demonstrated a high ratio of *P. acnes* detection (82%) in retinal biopsy specimen in patients with ocular sarcoidosis, whereas *P. acnes* was not detected (0%) in the control group. This marked difference strongly indicated that *P. acnes* may be associated with sarcoid uveitis. To the best of our knowledge, this is the first study to detect *P. acnes* within the retinal granulomas in patients with ocular sarcoidosis.

Many Japanese studies have supported the potential of *P. acnes* as a promising etiological agent of sarcoidosis^15–19^. Unlike tissues in contact with external air, such as the lungs and skin, the normal intraocular environment is completely aseptic and does not contain indigenous bacteria. On the other hand, *P. acnes* is the most common commensal bacterium in the peripheral lung tissue and mediastinal lymph nodes identified in subjects, even those without sarcoidosis. Indeed, in a previous study, *P. acnes* was isolated from half of 43 lungs and 8 of 11 mediastinal lymph nodes, mostly in pure culture. Moreover, the strains of *P. acnes* isolated from sarcoid lymph nodes were not specific to sarcoidosis^33^. Even IHC with PAB antibody, the same method as used in our current study, revealed the presence of *P. acnes* in 18% of surgically obtained samples and 22% of lymph node samples in patients without sarcoidosis^19^. Given that the retina is an aseptic environment, the presence of *P. acnes* within the retina is extremely unusual. In fact, no *P. acnes* was detected within retinas in the control group in the current study. Therefore, detecting *P. acnes* within retinas is a critical finding for confirming the role of *P. acnes* as an etiological agent of sarcoidosis.

Sarcoidosis commonly occurs between the ages of 20 to 30 and 50 to 60 years. In the present study, young patients with sarcoidosis were not included, because the patients who needed surgery were older than the average age for sarcoidosis. Further studies are needed to confirm that *P. acnes* is associated with sarcoid uveitis in young patients.

The detection frequency of *P. acnes* using PAB antibody may depend on the sizes of samples in lung granulomas; the detection frequency in surgically obtained lung samples was reported to be 74%, whereas that in lung biopsy samples was only 48%^19^. Surprisingly, the detection rate of *P. acnes* in retinal samples, which are extremely small, was 82%, which is more than that in surgically obtained comparatively large lung samples (74%). One possible explanation for the higher detection frequency of *P. acnes* within retinal granulomas than in lung granulomas is the lower grade of *P. acnes* degradation in the retina compared with that in the lung. Negi *et al*. suggested that the failure to detect *P. acnes* within lung granulomas of some sarcoid samples may be explained by the complete degradation of *P. acnes* antigens by granuloma cells^19^. This hypothesis is supported by the findings that PAB antibody reactivity was observed more frequently in immature granulomas than in mature granulomas, showing that the *P. acnes* antigen in lung granuloma cells may have been degraded and abolished as the granuloma matured. Because the respiratory tract is always in contact with external air, the function to remove and degrade pathogens in the respiratory track is innately developed. In contrast, intraocular tissue is not normally exposed to any pathogens. Under these specific conditions, *P. acnes* may be degraded more slowly in intraocular granulomas than in lung granulomas, thus leading to higher detection of *P. acnes*, even within extremely small retinal samples.

In the present study, retinal granulomas commonly contained small-sized and/or large-sized forms of *P. acnes*, and occasionally contained a few large spheroidal bodies detected with PAB antibody. These large spheroidal bodies were previously identified as Hamazaki-Wesenberg (HW) bodies in lung and lymph node samples^19^. HW bodies were initially found in lymph nodes as a nonspecific but frequently seen feature in sarcoidosis^34–36^. HW bodies are currently thought to be cell-wall-deficient *P. acnes* ^19,37,38^. We also think that these large spheroidal bodies in retinal samples in the present study are identical to the HW bodies seen in lung or lymph node samples, because of the similarity of sizes, shapes, and staining pattern.

If *P*. *acnes* is associated with ocular sarcoidosis, then why were all retinas not positive for *P. acnes* in the present study? One explanation is the size of the biopsied retinas. The biopsied samples must be extremely small because they should ideally be collected from the narrow areas of invisible retina. It may occur when one granuloma within an extremely small retinal sample is negative for *P. acnes*; the sample may then be considered negative, even if other granulomas react with the anti-*P. acnes* antibody. Alternatively, there may be other causes of ocular sarcoidosis. Indeed, Ishige *et al*. revealed that many *P. acnes* genomes were found in 80% (12/15) of lung lymph node samples from patients with sarcoidosis without coexistence of *P. granulosum* genomes, whereas in the remaining 20% (3/15), *P. granulosum* genomes were found without coexistence of *P. acnes*, suggesting that both bacteria may independently induce sarcoidosis^17^. In this present study, *P. acnes* was undetected in 2 of 11 (18%) of retinal sarcoid granulomas. In these patients, the failure to detect *P. acnes* may be explained by the presence of sarcoidosis arising from other pathogenic factors, such as *P. granulosum*, *Mycobacterium* species, or unknown environmental substances. Indeed, in the one of two patients, *M. intracellulare* was isolated from bronchoalveolar lavage fluid by bacterial culture, implying that the cause of sarcoidosis in this patient may be *M. intracellulare*.

As described above, *P. acnes* is not normally present in the retina. However, the mechanism through which *P. acnes* enters the intraocular tissue is not clear. In the present study, *P. acnes* was mainly detected in the granulomas in the inner retinal layer, where retinal vessels are mainly distributed. Moreover, inflammatory cells were infiltrated conspicuously in the perivascular area. Furthermore, large granulomas were detected in both the inner and outer retinal layers in some severe cases; however, there were no cases with granuloma presenting only in the outer retinal layer. Taken together, these findings implied that granulomas were caused by hematogenous transmission of *P. acnes*.

Sarcoidosis is characterized by an increased number of activated macrophage and CD4+ T cells and is therefore thought to be a Th1-driven inflammatory process^39,40^. In fact, we have previously reported that high CD4/CD8 ratios in vitreous-infiltrating lymphocytes were detected, and Th-1-related cytokines were upregulated in vitreous samples from patients with ocular sarcoidosis^41,42^. In the present study, retinal granulomas were comprised of T lymphocytes and macrophages. Because these inflammatory cells produce Th-1-related cytokines and can induce retinitis and vitritis, our findings are consistent with previous studies showing that sarcoidosis is caused by a Th1-driven inflammatory process.

Currently, the treatment of ocular sarcoidosis mainly involves local and additional systemic steroids. Although these treatments can successfully reduce the inflammation caused by sarcoidosis on clinical examination, they only treat inflammatory responses as symptomatic therapy. However, identification of the etiologic agent as *P. acnes* may facilitate the development of novel treatments for ocular sarcoidosis, i.e., a combination of curative treatment, such as antibiotics to reduce *P. acnes*, and symptomatic therapy.

Our retinal biopsy procedure was performed safely with no complications in all cases. Moreover, the mean best-corrected visual acuity improved significantly after the PPV.

In conclusion, our findings suggested the possibility that sarcoid granulomas are induced by the Th1 immune response against *P. acnes*, characterized by accumulation of CD68+ macrophage and CD4+ T lymphocytes, and that *P. acnes* may be hematogenously transmitted from the lung or another location to the retina in individuals with hereditary or acquired abnormalities in the immune system. Thus, *P. acnes* is associated with sarcoid uveitis. Because the retina is typically an aseptic organ, the high frequency of *P. acnes* in this study provides important insights into the pathogenesis of sarcoidosis. Further studies are needed to determine the precise mechanisms through which sarcoidosis is developed, particularly to overcome the limitation of the small sample size in this study.

## Methods

This was a prospective, case-control study performed in accordance with the tenets of the Declaration of Helsinki. All procedures were approved by the Institutional Review Board of the Kyoto Prefectural University of Medicine Hospital (# RBMR-C-864). The trial was registered in the University Hospital Medical Information Network Clinical Trial Registry. Written informed consent was obtained from all participants before enrollment.

### Participants

A total 24 eyes of 23 patients were enrolled in this study. All patients were Japanese. Of those 24 eyes, 12 eyes of 11 patients were diagnosed with biopsy-proven sarcoidosis. Of the 11 patients with sarcoidosis, six were also proven positive histopathological manifestations in organs other than eye by skin biopsy (two patients), transbronchial lung biopsy (two patient), and transbronchial needle aspiration of lymph nodes in the mediastinum (two patients) after retinal biopsy. These biopsies were performed at the time the skin, lung, and lymph node lesions appeared. The other five patients were comprehensively diagnosed by retinal biopsy and clinical examinations according to the statement on sarcoidosis of the ATS/ERS/WASOG^2^. As non-sarcoidosis controls, eight patients with rhegmatogenous retinal detachment (RRD), two patients with retinal detachment due to non-sarcoid uveitis (acute retinal necrosis and ocular toxoplasmosis), and two patients with vitreoretinal lymphoma were enrolled. The mean patient ages were 69.54 ± 4.50 years (range: 63–75 years) in the sarcoidosis group and 61.73 ± 10.01 years (range: 45–77 years) in the control group. In the sarcoidosis group, all cases had multiple retinal exudates located inferiorly in the fundus. All cases had severe vitreous opacities that were likely to cause decreased visual acuity.

### Sample collection procedure

For sample collection, 25-gauge pars plana vitrectomy was performed using a Constellation Vision System (Alcon Laboratories, Inc., Fort Worth, TX, USA) under a wide-angle viewing system. In the sarcoidosis group, local retinal detachment was manually created by infusing a balanced salt solution (Alcon Laboratories, Inc.) with a subretinal needle attached to a syringe. Retinectomy was performed using a bimanual technique with vitreoretinal forceps and curved scissors. Retinal samples were then collected using an indwelling needle. The nasal lower or temporal lower part of the retina with exudates was selected for collecting retinal samples. Retinal biopsies were safely performed without intraoperative complications in all cases. All collected retinas could be biopsied from the peripheral region to minimize postoperative visual field loss. In patients with RRD, a retinal flap from the retinal tear was obtained by retinectomy, as described above, performed during vitrectomy. In patients with acute retinal necrosis and ocular toxoplasmosis, detached retinas to be excised due to inflammation were collected. In patients with vitreoretinal lymphoma, retinas with intraretinal lesions were collected.

### PAB antibody

The PAB monoclonal antibody was generated in mice against whole-bacterial lysates of *P. acnes* according to previously reported methods^19^. In brief, BALB/c mice (CLEA Japan, Tokyo, Japan) were immunized with the sonicated whole-bacterial lysate of *P. acnes* (ATCC 11828). Hybridoma cell lines producing anti-*P. acnes* antibodies were checked by enzyme-linked immunosorbent assay with the bacterial lysate. Hybridoma cell lines with positive results were screened by IHC with formalin-fixed and paraffin-embedded tissue sections of the *P. acnes*-injected rat liver, which was obtained by intravenous injection of 30 mg of heat-killed *P. acnes* into female Sprague–Dawley rats (CLEA Japan) 1 h before killing the rat. Similarly-prepared liver tissue sections of rats injected with each strain of other control bacteria were also examined to confirm the specificity of the antibody to *P. acnes*. Hybridoma cell lines producing the antibody that generated a strong reaction specific to *P. acnes* on rat liver sections were selected and further screened by IHC with formalin-fixed and paraffin-embedded tissue sections of the sarcoid lymph nodes, in which a large number of *P. acnes* genomes were detected. The hybridoma producing the antibody that generated the most specific reaction product on human tissue sections was selected and cloned by two rounds of limiting dilution. A single hybridoma clone was then implanted into the intraperitoneal space of severe combined immunodeficiency mice (CLEA Japan). At 1 or 2 weeks after implantation, ascites was collected and used as an undiluted antibody without further purification.

The specificity of this monoclonal antibody was previously described based on the results obtained with IHC and western blot analysis^19^. The PAB antibody reacted with all strains of *P. acnes*, irrespective of the serotype, strain type, or clinical isolate, and there was no cross-reactivity with other propionibacteria including *P. granulosum* and *P. avidum* or other control bacteria. The PAB antibody recognizes lipoteichoid acid because it was found to react with the lipoteichoic acid purified from *P. acnes*. It is unlikely that the purified fraction of lipoteichoic acid was contaminated with another structure such as lipoglycan, because the band on the western blot membrane was slightly broad but nevertheless clearly visible as a single band. Reaction products of PAB antibody were localized at the peripheral area of the bacterial body, consistent with the localization of cell-membrane-bound lipoteichoic acid (see Supplemental Fig. 1).

### Histological and immunohistochemical analysis

Biopsied retinal samples from the sarcoidosis group and corresponding control samples were examined. Sections were fixed in Zamboni solution, embedded in paraffin, cut, and mounted on silane-coated slides. Deparaffinized sections were stained with hematoxylin-eosin (HE). Adjacent sections were also examined by IHC as described previously^19^. We confirmed beforehand that Zamboni solution does not alter the IHC staining specificity of the PAB antibody. After the sections were deparaffinized and rehydrated, they were microwaved (Microwave Processor H2850; Energy Beam Sciences, East Granby, CT, USA) in 10 mmol/l citrate buffer (pH 6.0) for 40 min at 97 °C. The sections were then treated with 3% hydrogen peroxide in methanol for 10 min. The sections were first incubated with normal horse serum (Vectastain Universal Elite ABC Kit; Vector Laboratories, Burlingame, CA, USA) and then incubated overnight at room temperature with the diluted PAB antibody (1:15,000) in a humidified chamber. The sections were further incubated for 30 min with the biotinylated secondary antibody, followed by a 30-min incubation with streptavidin-peroxidase complex (Vectastain Universal Elite ABC Kit), at room temperature. Before and after each step, the sections were washed in phosphate-buffered saline containing 0.5% Tween-20. The signal was developed as a brown reaction product using the peroxidase substrate diaminobenzidine (Histofine Simplestain DAB Solution; Nichirei Bioscience, Tokyo, Japan). Anti-human CD4 mouse monoclonal antibodies (clone 4B12; Novocastra, Newcastle upon Tyne, United Kingdom), anti-human CD8 mouse monoclonal antibodies (clone 1A5; Novocastra), and anti-human macrophage CD68 mouse monoclonal antibodies (clone KP1; DAKO, Glostrup, Denmark) were also used. All sections were counterstained with hematoxylin.

### Statistical analysis

Fisher’s exact test was used to evaluate statistical differences between groups. Difference with a *p* value of less than 0.01 was considered significant.

### Data Availability

The datasets generated during and/or analysed during the current study are available from the corresponding author on reasonable request.

## Electronic supplementary material

Supplemantal Figure 1
