# Supplementary material for: Immunohistochemical Detection of Propionibacterium acnes in the Retinal Granulomas in Patients with Ocular Sarcoidosis
Source: Sci Rep. 2017 Nov 9;7:15226. doi: 10.1038/s41598-017-15710-0 (PMC5680245; doi:10.1038/s41598-017-15710-0)
Supplement: Supplementary file 1 — Supplemantal Figure 1 [file 41598_2017_15710_MOESM1_ESM.pdf]

Title: Immunohistochemical Detection of *Propionibacterium acnes* in the Retinal Granulomas in Patients with Ocular Sarcoidosis

Authors: Kenji Nagata*, Yoshinobu Eishi, Keisuke Uchida, Kazuhito Yoneda, Hiroki Hatanaka, Toru Yasuhara, Maho Nagata, Chie Sotozono and Shigeru Kinoshita

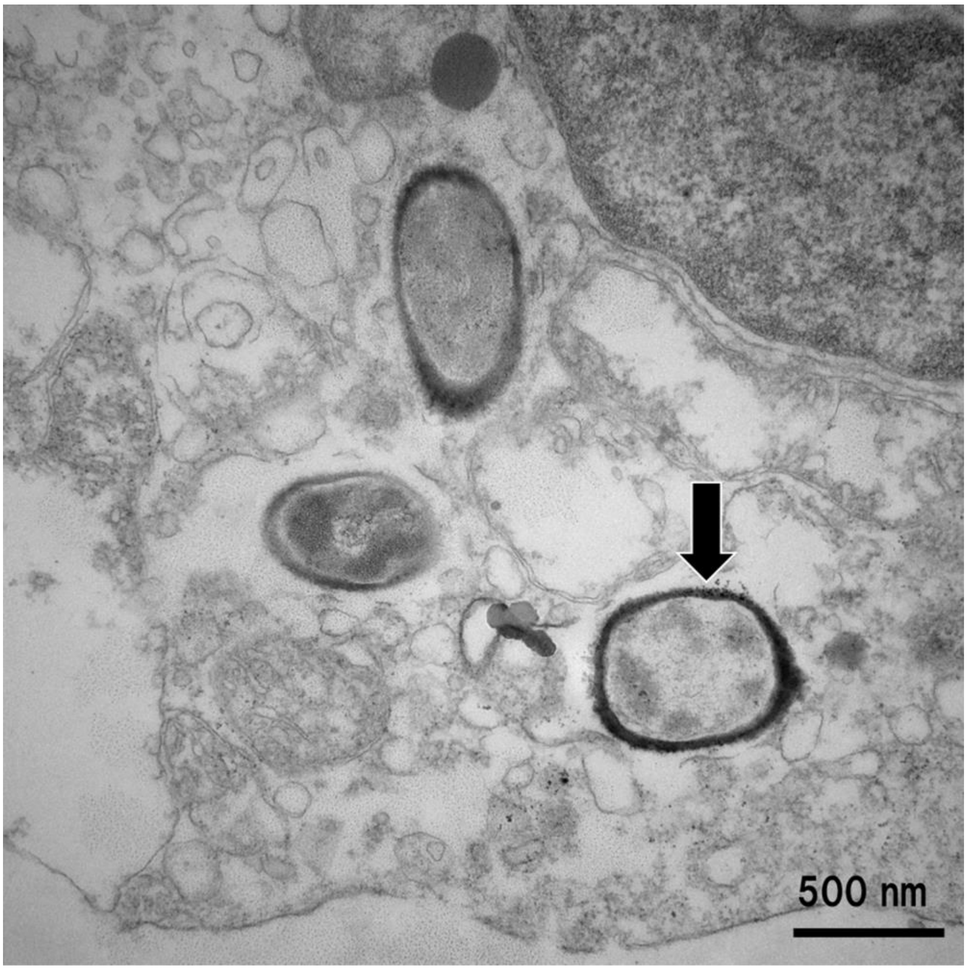

Supplemental Figure 1: Electron-microscopic localization of the antigen detected by PAB antibody

Cultured cells of human monocyte (THP-1) were infected by P. acnes and IHC with PAB antibody was performed at 6 hours postinfection. Reaction products of peroxidase substrate diaminobenzidine are localized at the peripheral area of the bacterial body (indicated by an arrow) consistent with localization of cell-membrane-bound lipoteichoic acid.
